# IQ and Internalising Symptoms in Adolescents with ASD

**DOI:** 10.1007/s10803-020-04810-y

**Published:** 2020-12-30

**Authors:** Monisha Edirisooriya, Dominika Dykiert, Bonnie Auyeung

**Affiliations:** 1 University of Edinburgh, School of Philosophy, Psychology and Language Sciences, University of Edinburgh Medical School, Edinburgh, Scotland UK; 2 Guy’s & St Thomas’ NHS Foundation Trust, Westminster Bridge Road, SE1 7EH London, England; 3 University of Edinburgh, School of Philosophy, Psychology and Language Sciences, University of Edinburgh, Centre for Cognitive Ageing and Cognitive Epidemiology, Edinburgh, Scotland, UK; 4 Anna Freud Centre for Children and Families, The Kantor Centre of Excellence, 4-8 Rodney Street, London, N1 9JH England; 5 University of Edinburgh, School of Philosophy, Psychology and Language Sciences, Room S30, Psychology Building, 7 George Square, Edinburgh, EH8 9JZ Scotland UK; 6 Autism Research Centre, University of Cambridge, Cambridge, England

**Keywords:** Autism spectrum disorder, Adolescents, Anxiety, Depression, Intelligence quotient, Internalising symptoms

## Abstract

Intelligence quotient (IQ), has been found to relate to the presence of internalising symptoms in autism spectrum disorder (ASD). This meta-analysis sought to clarify the direction of the relationship between IQ and two prevalent internalising symptoms, anxiety and depression, in adolescents with ASD. Secondly, this study aimed to highlight methodological factors contributing to inconsistent findings in existing research. Self-reported anxiety was found to be significantly higher in youth with a lower IQ, while depression was positively associated with IQ. Consequently, parents, schools and clinicians should be cautious of underestimating anxiety in youth with a lower IQ. However, care should also be taken to ensure adolescents with ASD without intellectual disabilities are not overlooked with regards to social and emotional support.

As awareness of both autism spectrum disorder (ASD) and mental illness has progressed, they have become increasingly discussed in the academic and public sphere. This study seeks to further the understanding of the simultaneous presence of these conditions. Such knowledge is essential to improving the mental health of individuals with ASD, a current priority for the ASD community (Povey 2016).

ASD encompasses a ‘spectrum’ of neurodevelopmental disabilities, replacing the separate diagnostic labels of Asperger syndrome, autistic disorder and pervasive developmental disorder not otherwise specified. To differing extents, functions such as socialisation, flexible thinking and communication are affected (American Psychiatric Association 2013). These deficits have been linked to a host of psychiatric disorders, estimated to affect 70% of youth with ASD (Simonoff et al. 2008). They include internalising symptoms: emotional problems developed and largely maintained within the individual, such as depression, anxiety, fear, and low self-esteem (Merrell 2008; Ollendick and King 1994). It has been highlighted that depression and anxiety are particularly prevalent in individuals with ASD (Ghaziuddin et al. 2002; van Steensel et al. 2011).

Adolescence is known to be a period of vulnerability to depression and anxiety and this is no exception within the ASD population (Scherff et al. 2014). Anxiety is a dominant presenting problem for adolescents with ASD (Williams et al. 2015). Furthermore, research has found increased rates of anxiety in adolescents with ASD, compared to the general population (Vasa et al. 2013). In addition, adolescence is a time when youth transition between health service providers. A common fear for parents of youth with autism is that their children will be under-supported during this time (Weiss 2008); both child and adult services must be well equipped to provide mental health support for young people with autism. Moreover, psychiatric and behavioural disorders in youth with ASD have been shown to result in higher medical expenditure compared to those without ASD, which may cause caregiver-stress (McCarthy 2007). Hence, enhancing the knowledge and understanding of internalising symptoms in individuals with ASD during adolescence is valuable. It may aid parents, educational and health services in identifying those at higher risk and implementing prevention strategies, as well as adequately managing those affected. Addressing mental illness effectively during this period is vital, since it can benefit school performance, social development, and behavioural outcomes in adulthood (McEwan et al. 2007; Murphy et al. 2015).

Research concerning internalising symptoms in people with ASD has highlighted intelligence as an important variable. Intelligence is defined by intelligent quotient (IQ) for the purpose of this study, with intellectual disability defining an IQ score of below 70 (Luckasson et al. 2002). However, evidence regarding the correlation between IQ and depression or anxiety in individuals with ASD is conflicting. Negative, positive, non-linear and non-existent associations have all been reported, as outlined below.

## Negative Correlation Between IQ and Internalising Symptoms

Prior studies have suggested that lower IQ relates to higher levels of internalising symptoms in young people with ASD (Amr et al. 2012; Rosa et al. 2016). Similarly, a previous meta-analysis found a negative correlation between IQ and anxiety in children and adolescents with ASD (van Steensel et al. 2011).

Some explanations for this negative association have been suggested. A lower IQ could cause poorer coping strategies, so that unfamiliar tasks induce anxiety (Crabbe 2001). Given this, a lower IQ may correlate with more emotional problems through adolescence, a time of unfamiliar experiences. By contrast, a higher IQ might allow for adaptive coping skills, such as seeking help when needed, shown to be associated with lower depression levels in youth with ASD (Pouw et al. 2013).

A poorer ability to communicate feelings could also be a factor in the negative correlation between IQ and internalising symptoms. Difficulties in identifying and describing feelings are involved in the personality trait alexithymia (Taylor et al. 1991). Milosavljevic et al. (2016) found that a lower verbal IQ was associated with higher levels of alexithymia in adolescents with ASD. Presence of alexithymia was also related to increased anxiety. Additionally, communication deficits associated with a lower IQ have been suggested to cause problems with conversing and dismissing fears (Pickersgill et al. 1994). Overall, difficulties in expressing feelings could result in individuals keeping their problems internalised and receiving inadequate support. Both of these factors may explain the observation of higher internalising symptoms in less intelligent youth with ASD.

## Positive Correlation Between IQ and Internalising Symptoms

Conversely, several studies have reported a link between a higher IQ and depression in people with ASD (Fung et al. 2015; Ghaziuddin and Greden 1998; Greenlee et al. 2016; Mayes et al. 2011a, b). A positive correlation between IQ and anxiety has also been observed (Gotham et al. 2015; Mayes et al. 2011a, b; Niditch et al. 2012; Sukhodolsky et al. 2008).

Two explanations arise from this research. The first explanation is that higher intelligence may indeed increase the risk for internalising symptoms in individuals with ASD. Several underlying mechanisms have been proposed; for instance, a lack of social support. Socialising is known to improve emotional health (Kawachi and Berkman 2001). Taylor and Seltzer (2011) compared the post-secondary school activities of youth with ASD during the transition to adulthood, with and without an intellectual disability. Those without an intellectual disability were 3 times more likely to have no daytime activities. Eighty-six percent of participants without daytime activities had a comorbid psychiatric diagnosis, including anxiety or depression. One reason for a lack of social support may be that young people without significant intellectual disability are more likely to attend educational institutions with typically developing peers and the number of such pupils is increasing (Humphrey and Symes 2010). Compared to institutions that care for young people with disabilities, these institutions may place less emphasis on tailored social and emotional support. Furthermore, youth with ASD are at an increased risk of social exclusion by their school peers, which may also negatively impact emotional health (Humphrey and Symes 2010). Consequently, an inadequacy of social support for more intelligent youth could be one factor causing greater emotional difficulties.

Another proposed mechanism is that youth with a higher IQ may have greater social understanding, including an increased awareness of their own social competence and deficits, which may be emphasised to themselves when attending mainstream educational institutions*.* This has been suggested to cause anxiety and depression (Caamaño et al. 2013; Kushki et al. 2013; Vickerstaff et al. 2007). In addition, an enhanced awareness of personal deficits in the presence of a higher IQ could increase vulnerability to low self-esteem. In line with this suggestion, lower self-esteem has been observed in children with autism with an IQ of 80 or above, compared to those with an IQ below 80 (Mayes et al. 2011a, b). Although an internalising symptom itself, low self-esteem could also predispose to anxiety and depression.

The second, alternative explanation is that a higher IQ may provide individuals with greater means to report their emotional difficulties, over those with a lower IQ (Caamaño et al. 2013). Moreover, in less intelligent individuals who cannot express their problems, anxiety symptoms may be mistaken for communication deficits and resultantly underestimated (Davis et al. 2011). The ability to report symptoms may therefore confound the relationship between IQ and internalising symptoms.

## Non-Linear and No Correlation Between IQ and Internalising Symptoms

To add to this discrepancy in findings, a previous meta-analysis suggested a quadratic relationship, such that youth with an IQ between 70–87 experience higher anxiety than others. However, tentative results meant further investigation was necessary to conclude this relationship (van Steensel et al. 2011). A subsequent study could not find a quadratic or a linear correlation between IQ and anxiety (Eussen et al. 2012). Others have also reported no association between IQ and internalising symptoms (Gotham et al. 2015; Simonoff et al. 2008; Strang et al. 2012). The most obvious explanation is that IQ is unrelated to internalising symptoms in individuals with ASD. However, methodological factors could be obscuring a significant correlation, some of which are suggested below.

## Factors Contributing to Conflicting Findings

Using IQ as an index of intelligence could affect the correlation found with internalising symptoms in the ASD population: IQ tests can underestimate intelligence in those with ASD (Nader et al. 2016). The IQ range used in studies could also impact the association found with internalising symptoms depending on whether higher or lower IQs are included. Individual studies, as well as the meta-analysis by van Steensel et al. (2011), have reported restricted sample IQ ranges as a limitation (Eussen et al. 2012; Strang et al. 2012).

The psychometric instrument used to measure internalising symptoms could also influence the correlation with IQ. Instruments are based on differing criteria, for example they do not always correspond with Diagnostic and Statistic Manual of Mental Disorders (DSM) guidelines, limiting their comparability. Also, the majority of these measures are designed for typically developing youth (Vasa et al. 2013) and may vary in their ability to accurately assess internalising symptoms in people with ASD.

Existing studies suggest that grouping multiple internalising symptoms, or even subtypes of a symptom, into one variable could mask differing associations with IQ. Gotham et al. (2015) found a positive correlation between IQ and anxiety, but no association with depression. A previous meta-analysis found that the direction of the correlation between IQ and anxiety varied, depending on the anxiety subtype analysed (van Steensel et al. 2011).

In addition, psychiatric medication use within a sample may alter the level of internalising symptoms experienced and subsequently reported, thus affecting the correlation with IQ (Mazefsky et al. 2011), given that such medication aims to lessen anxiety and depression.

Varying accuracy both within and between self and caregiver-reported internalising symptoms may also impact findings. It is currently debatable as to whether caregiver-reports provide reliable depictions of internalising symptoms in youth with ASD and further evidence is required. Indeed, a limitation of the previous meta-analysis by Van Steensel et al (2011) was the insufficient data to test whether informant (parent versus child) moderated the relationship between anxiety and ASD. They called for future research to test how anxiety frequency varies across informants. On the one hand, parents could underestimate emotional difficulties in some children (Cosi et al. 2010; Muris et al. 1999). Capps and colleagues found that parents observed less sadness and fear in children who reported lower perceived self-worth, compared to children with higher self-worth. It is possible that more intelligent youth are able to engage in greater social interaction. While this may highlight their deficits and resultantly lower their self-worth, parents may only perceive positive social adjustment (Capps et al. 1995). On the other hand, parental anxiety can cause over-reporting of anxious symptoms in children (Bernstein et al. 2005). Nevertheless, there is evidence that parent-rated anxiety and depression are effective methods of assessment and comparable to self-reports (Ozsivadjian et al. 2014; Sukhodolsky et al. 2008). As mentioned previously, youth with higher IQs may be more able to report their symptoms than those with lower intelligence. Additionally, research shows that fewer parent–child discrepancies of anxiety symptoms occur when children have a higher verbal IQ (Ooi et al. 2016). It has been suggested that using both parent and self-reports would provide a more representative measurement of internalising symptoms experienced by an individual with ASD (Ooi et al. 2016).

Finally, gender distributions and ages of samples may affect levels of internalising symptoms measured, and consequently the correlation found with IQ in individuals with ASD. However, evidence pertaining to these factors is inconsistent. While some findings show no relationship between gender and depression or anxiety in people with ASD (Mayes et al. 2011a, b), Solomon and colleagues reported that female adolescents had higher internalising symptoms than males (Solomon et al. 2012). Also, anxiety and depression have been shown to increase as children with ASD grow older (Mayes et al. 2011a, b), but other research suggests there is no relationship between age and these symptoms (Strang et al. 2012). By focusing on adolescence, the current study aims to limit any significant effects of younger versus older age on the relationship between IQ and internalising symptoms.

## Rationale and Aims

Ultimately, anxiety and depression are known to be common in both autism and adolescent populations; yet the relationship between IQ and these internalising symptoms in individuals with ASD is not well understood. This meta-analysis seeks to contribute towards a better understanding of this relationship, in order to allow affected and high-risk youth to be more readily identified and supported in clinical practice, educational settings and at home. Furthermore, young people with better mental health outcomes are more likely to be able to positively contribute to society as adults.

Thus, the primary aim of this study is to synthesise the current evidence to distinguish the overall direction of the association between IQ and internalising symptoms in adolescents with ASD. Secondly, this study aims to highlight methodological factors contributing to inconsistent findings in the existing literature, in order to assist in achieving the primary aim, as well as guide future research.

Anxiety and depression are investigated as separate outcomes in this meta-analysis to account for differing associations with IQ. Additionally, high rates of internalising disorders have been shown not only in individuals clinically diagnosed with ASD, but also in the broader population with ASD traits (Simonoff et al. 2008). This study therefore investigates youth across the autism spectrum, with or without a clinical diagnosis.

## Methods

This meta-analysis was completed following the Meta-analysis Of Observational Studies in Epidemiology (MOOSE) guidelines (Stroup et al. 2000).

### Systematic Literature Search Strategy

An expert in systematic literature searching approved the following search strategy. The databases MEDLINE, PsycINFO and Scopus were used to identify studies for this investigation. Search terms were selected, pertaining to four subjects: intelligence, internalising symptoms, ASD and adolescence. These four groups of search terms were separated by the Boolean operator ‘and’, while ‘or’ was used to separate terms within each group. Resultantly, studies relating to all four groups were returned. Studies from the earliest record date of each database to 10/02/2017 were obtained. Appendix A contains the full list of search terms.

### Eligibility Criteria and Study Selection Process

Specific eligibility criteria guided the selection of studies. Publications had to be empirical studies from peer-reviewed journals published in English. All other types of literature were excluded, such as books, book reviews, dissertations, meta-analyses and literature reviews.

Studies had to include an ASD sample. A finding for this sample on the association between IQ and internalising symptoms (anxiety and/or depression) was also required, with or without an effect size. The internalising symptoms could be subclinical or clinical.

Due to a lack of existing studies focusing solely on adolescence, it was determined that the sample needed a mean age between 10.0–19.0 years at the time of assessment, in order to be defined as adolescent (WHO 2017) and included in the study. In some studies, data on IQ or internalising symptoms was missing for a minority of participants and there was no corrected mean age for the subset of the sample with data. The study was then included if it was deemed to be likely that the mean age would fall within 10.0–19.0 years, based on the age information given and extent of missing data.

Following the above criteria, adolescent age, English language, human population and peer-reviewed journal article restrictions were imposed on searches within the databases where possible, to minimise manual exclusion of the retrieved studies. It was also outlined that if two or more studies used overlapping cohorts of participants, the article with the larger sample size or more necessary data reported would be included.

The publications received from the databases were managed using Endnote. Duplicate publications were removed electronically and any remaining duplicates were deleted manually. Next, the specified eligibility criteria were applied to the titles and abstracts of the 672 non-duplicated studies. Full text articles were then obtained for a) studies that met all eligibility criteria b) studies that could not be shown to meet or fail eligibility criteria based on the title and abstract alone, to allow closer evaluation.

The authors of this study independently tested the publications against the eligibility criteria. Where there was uncertainty about whether a paper met the criteria for inclusion, all three researchers discussed the paper until a decision was reached.

Efforts were made to ensure systematic inclusion of all relevant existing studies. The reference lists of the 167 full text articles were examined and five additional studies were identified that met the eligibility criteria. Furthermore, two studies were located from the 2016/17 issues of two reoccurring journals from the systematic literature search: *Autism* and *Journal of Autism and Developmental Disorders*. The rationale for looking at these issues was based on newer articles not necessarily having been assigned Medical Subject Headings in the databases, increasing their risk of being missed in the search process.

From the 28 articles that met the eligibility criteria, 15 studies were included in the meta-analysis, as they reported an effect size (whether significant or non-significant) for the relationship between IQ and depression/anxiety. Thirteen studies were excluded from the meta-analysis for three reasons. Firstly, studies that investigated anxiety and depression as separate entities, but did not report an effect size or statistical information that could be used to calculate an effect size, were excluded. Secondly, the study by Hallett et al. (2013) was excluded, because the analysis software did not accept intraclass correlations. Thirdly, studies that grouped anxiety and depression together, with or without other symptoms, were excluded.

These 13 studies were included as appropriate in an anxiety, depression, or grouped internalising symptoms vote-counting procedure instead. Vote-counting involved totalling the number of studies reporting a positive, negative or no association between IQ and the internalising symptom(s). Although the current study set out to investigate anxiety and depression separately, articles that grouped internalising symptoms together were included in the vote-counting process to test the theory that the correlation is unclear when multiple symptoms are assessed as one variable. A meta-analysis was not eligible to test this, because only two studies reported effect sizes and the symptoms grouped together differed between the two studies.

**Fig. 1:**
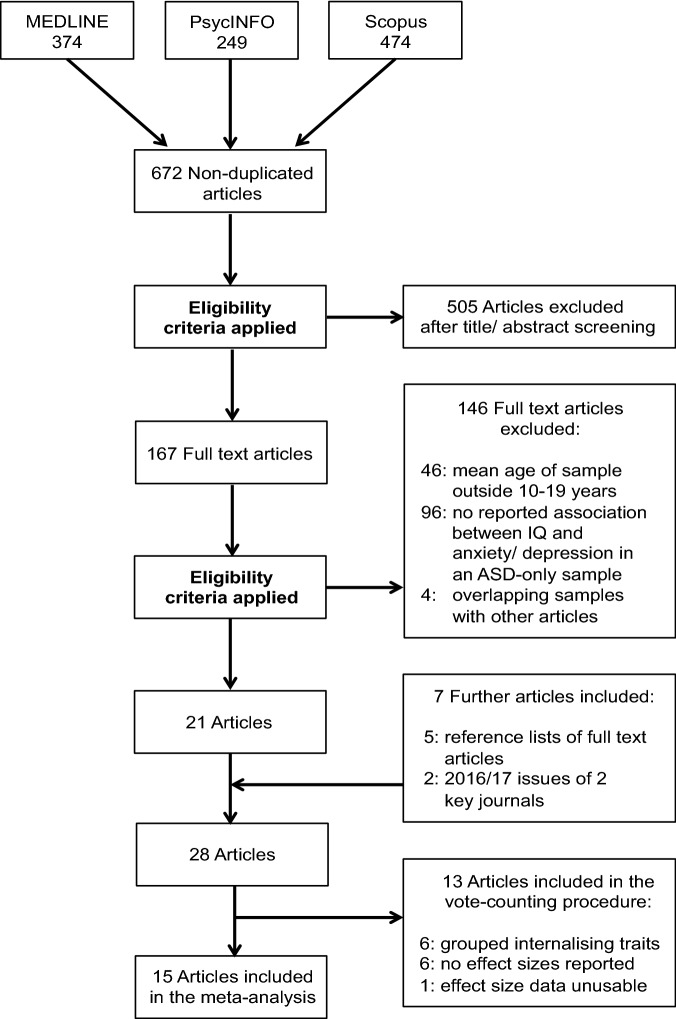
Flow chart showing the systematic literature search and study selection process

Figure 1 summarises the literature search and study selection process. The study characteristics of the 13 studies excluded from the meta-analysis are detailed in Table 1.

**Table 1:** Studies excluded from the meta-analysis and included in the vote-counting procedure

Study	N	Gender % male	Mean age in years (range)	Autism type (assessment)	IQ test (score)	IQ range	Internalising symptom (instrument)
Bitsika et al. 2016	150	100	11.2 (6–18)	ASD (DSM-V)	WASI-II (FSIQ)	73–132	Grouped (CASI)
Gjevik et al. 2011^a^	71	82	11.8 (6–18)	ASD (ADI-R, DSM-IV)	Leiter-R (NVIQ)	30–129	Anxiety, Depression (K-SADS-PL)
Gotham et al. 2013	1429	86	10.2 (6–18)	ASD (ADI-R, ADOS)	SSC (VIQ)	5–167	Anxiety (CBCL)
Hallett et al. 2013	141	85	13.5 (10–15)	ASD (ADI-R, ADOS, DAWBA)	WASI (IQ)	50-130^c^	Anxiety (RCADS)
Kerns et al. 2014	59	78	10.5 (7–17)	ASD (ADI-R, ADOS)	DAS-II, WISC-IV (IQ)	67–158	Anxiety (ADIS-C/P, BASC-2, NASSQ, SCARED)
Kim et al. 2000	59	88	12.0 (9–14)	PDD (ADI)	Leiter, Stanford-Binet-IV (IQ)	> 68	Anxiety (OCHS-R)
Mazzone et al. 2013	30	100	11.1 (7–16)	AS, HFA (ADOS-G, DSM-IV-TR)	WISC-III (FSIQ)	≥ 85	Depression (CBCL, CDI, CDRS-R)
Meyer et al. 2006	31	84	10.1 (8–14)	AS (ASSQ, ASAS)	WISC-III (IQ)	NA	Grouped (BASC)
Rosa et al. 2016^b^	50	92	12.0 (7–17)	HFASD (ADI-R)	WASI-III, WISC-IV (IQ)	≥ 70	Grouped (K-SADS-PL)
Schroeder et al. 2011^a^	15	NA	12.1 (6–16)	AS (DSM-IV, KADI)	WASI (FSIQ)	89–141	Grouped (CBCL)
Taylor and Henninger 2015	39	80	18.7 (17–22)	ASD (ADI-R, ADOS)	Stanford-Binet (IQ)	40–137	Grouped (RHSS)
van Steensel et al. 2013	40	90	11.1 (8–18)	ASD (DSM-IV)	WISC-R (IQ)	50-130^d^	Grouped (KID-SCID)
Vickerstaff et al. 2007	22	86	11.9 (8–14)	HFASD (DISCO)	WASI (FSIQ)	82–141	Depression (BASC, CDI)

N = Autism sample size, IQ = Intelligence Quotient

^a^Obtained from reference lists of the 167 full text articles

^b^Obtained from key journal: *Autism*

*NA* Information not available, *ASD* Autism Spectrum Disorder, *PDD* Pervasive Developmental Disorder, *AS* Asperger Syndrome, *HFA* High Functioning Autism, *HFASD* High Functioning Autism Spectrum Disorder

DSM(-TR) = Diagnostic and Statistic Manual of Mental Disorders(-Text Revision), ADI(-R) = Autism Diagnostic Interview(-Revised), ADOS(-G) = Autism Diagnostic Observation Schedule(-Generic), DAWBA = Development And Well-Being Assessment, ASSQ = Autism Spectrum Screening Questionnaire, ASAS = Australian Scale for Asperger’s Syndrome, KADI = Krug Asperger’s Disorder Index, DISCO = Diagnostic Interview for Social and Communication Disorders

*WASI* Wechsler Abbreviated Scale of Intelligence, *SSC* Simons Simplex Collection instruments for cognitive testing- no further information given, *DAS* Differential Abilities Scale, *WISC(-R)* Wechsler Intelligence Scale for Children(-Revised), *FSIQ* Full-Scale Intelligence Quotient, *NVIQ* Non Verbal Intelligence Quotient, *VIQ* Verbal Intelligence Quotient

^c^11.5% participants with an IQ below 50 and 1.5% participants with an IQ above 130

^d^ 3 participants with an IQ above 130

Grouped = Anxiety and depression grouped as one variable—plus or minus other psychological problems

*CASI* Child and Adolescent Symptom Inventory, *K-SADS-PL* Kiddie Schedule for Affective Disorders and Schizophrenia for School-Aged Children–Present and Lifetime, *CBCL* Child Behaviour Checklist, *RCADS* Revised Children’s Anxiety and Depression Scale, *ADIS-C/P* Anxiety Disorders Interview Schedule-Child/Parent, *BASC* Behaviour Assessment System for Children, *NASSQ* Negative Affectivity Self-Statement Questionnaire, *SCARED* Screen for Anxiety and Related Emotional Disorders, *OCHS-R* Ontario Child Health Study-Revised, *CDI* Children’s Depression Inventory, *CDRS-R* Children’s Depression Rating Scale-Revised, *RHSS* Rochester Health Status Survey, *KID-SCID* Structured Clinical Interview for Diagnostic and Statistical Manual-Version Four Childhood Disorders

% Male rounded to whole percentage, mean ages rounded to one decimal place, age ranges rounded to nearest year

### Data Extraction

For each study included in the meta-analysis, the following information was extracted: study size; age and gender distribution of the ASD sample; IQ test, score and range; type of internalising symptom (anxiety/depression); method of assessing internalising symptom, and method of confirming ASD status. Any details on whether participants were on psychiatric medication were also recorded. Furthermore, the correlation coefficient for the association between IQ and anxiety or depression was extracted from each study. Where no correlation coefficient was reported, alternative statistics that could be used to calculate an effect size were recorded (Table 2). In order to fulfil the methodological criteria that analyses cannot contain more than one effect size per study, three studies required a decision to be made regarding which statistic to extract. Appendix B outlines the rationales for the choices made.

**Table 2:** Studies included in the meta-analysis

Study	N	Gender % male	Mean age in years (range)	Autism type (assessment)	IQ test (score)	IQ range	Internalising symptom—subtype (instrument)	Effect size data	Quality score (failed criterion)
de Bildt et al. 2005	67	NA MF	10.9 (4–18)	Autism (DSM-III)	WISC-R (IQ)	36–70	Anxiety—fear of changes (CSBQ)	Mean, SD, N	4 (C)
Gadow et al. 2016	214	81	10.4 (6–12)	ASD (CASI-4R, DSM-IV, ADOS)	NA (IQ)	≥ 70^c^	Depression (CASI-4R)	*r*	3 (C, D)
Ghaziuddin and Greden 1998	23	87	10.5 (≥ 7)	Autism/PDD (DSM-III-R)	NA (FSIQ)	≥ 50	Depression (DSM-III-R, Reiss scale)	Mean, SD, N	3 (C, D)
Hollocks et al. 2014	52	100	12.9 (10–16)	ASD (ADOS, ADI)	WASI (FSIQ)	76–138	Anxiety—any (CAPA)	Mean, SD, N	5
May et al. 2015	46	52	10.9 (7–12)	Autistic/AS (DSM-IV-TR)	WISC-IV (FSIQ)	> 70	Anxiety—sleep (CSHQ)	*r*	5
Mazefsky et al. 2011	38	82	12.0 (10–17)	Autistic/AS (ADOS-G, ADI-R)	WASI (IQ)	71–144	Anxiety—any (RCMAS)	*r*	5
Neil et al. 2016^a^	64	86	10.4 (6–15)	Autistic (ADOS-G, ADOS-2)	WASI-II (FSIQ)	70–129	Anxiety—any (SCAS-P)	*r*	4 (C)
Oswald et al. 2016^b^	32	56	14.9 (12–17)	ASD (AQ, ASDS)	KBIT-2 (FSIQ)	≥ 75	Anxiety—any, Depression (RCADS-P, CES-D)	*r*	4 (C)
Rodgers et al. 2012	67	87	11.2 (8–16)	ASD (ADOS, SCQ)	WASI (FSIQ)	NA	Anxiety—any (SCAS-P)	Mean, SD, N	4 (C)
Simonoff et al. 2008^a^	112	88	11.5 (10–14)	ASD (ADOS-G, ADI-R, ICD-10)	WISC-III (FSIQ)	19–124	Anxiety—any (CAPA)	OR	4 (C)
Strang et al. 2012	95	86	11.7 (6–18)	ASD (DSM-IV, ADOS, ADI-R)	WASI, WISC-IV (FSIQ)	71–144	Anxiety—any, Depression (CBCL)	t, N	5
Vasa et al. 2013	115	85	13.9 (12–17)	ASD (DSM-IV, ADOS)	Stanford-Binet-V (ABIQ)	NA	Anxiety—any (CBCL)	*r*	4 (C)
White and Roberson-Nay 2009	17	90	12.1 (7–14)	ASD (ADOS, SCQ)	NA (IQ)	NA	Anxiety—any (MASC)	t, N	4 (D)
Wigham et al. 2015^a^	49	89	12.5 (8–16)	ASD (SRS)	WASI (FSIQ)	≥ 70	Anxiety—any (SCAS-P)	*r*	4 (C)
Witwer and Lecavalier 2010	58	82	11.2 (6–17)	ASD (ADI-R)	Stanford-Binet-V (IQ)	42–150	Anxiety—GAD sub-score (P-ChIPS)	χ^2^, N	4 (C)

N = Autism sample size relating to the effect size reported, IQ = Intelligence Quotient

^a^Obtained from reference lists of 167 full text articles

^b^Obtained from key Journal of Autism and Developmental Disorders, *ASD* Autism Spectrum Disorder, *PDD* Pervasive Developmental Disorder, *AS* Asperger Syndrome, *NA* Information not available, *MF* Male and female participants, *DSM(-TR/R)* Diagnostic and Statistic Manual of Mental Disorders(-Text Revision/ Revised), *CASI-4R*

Child and Adolescent Symptom Inventory-4Revised, *ADOS(-G)* Autism Diagnostic Observation Schedule(-Generic), *ADI(-R)* Autism Diagnostic Interview(-Revised), *AQ* Autism-Spectrum Quotient-Adolescent Version, *ASDS* Asperger’s Syndrome Diagnostic Scale, *SCQ* Social Communication Questionnaire, *ICD* International Classification of Disease, *SRS* = Social Responsiveness Scale, *WISC(-R*) Wechsler Intelligence Scale for Children(-Revised), *WASI* Wechsler Abbreviated Scale of Intelligence, *KBIT* Kaufman Brief Intelligence Test, *FSIQ* Full-Scale Intelligence Quotient, *ABIQ* Abbreviated Battery Intelligence Quotient (reflects FSIQ), *c *IQ range for 75% of the sample, *GAD* Generalised Anxiety Disorder, *CSBQ* Children’s Social Behaviour Questionnaire, *CAPA* Child and Adolescent Psychiatric Assessment, *CSHQ* Children’s Sleep Habits Questionnaire, *RCMAS* Revised Children’s Manifest Anxiety Scale, *SCAS-P* Spence Children’s Anxiety Scale-Parent version, *RCADS-P* Revised Children’s Anxiety and Depression Scale-Parent version, *CES-D* Centre for Epidemiological Studies-Depression symptoms index, *CBCL* Child Behaviour Checklist, *MASC* Multidimensional Anxiety Scale for Children, *P-ChIPS* Children’s Interview for Psychiatric Syndromes-Parent version, *SD* = Standard Deviation, *r* Correlation coefficient, *t* Independent t-test value, *OR* Unadjusted odds ratio, *χ*^*2*^ Chi-squared value

% Male rounded to whole percentage, mean ages rounded to one decimal place, age ranges rounded to nearest year

### Quality Assessment

A novel 5-point quality assessment score was designed for this meta-analysis, a concept taken from Bhutta et al. (2002). The quality criteria incorporated factors likely to influence the association between IQ and anxiety or depression, based on examination of existing literature.

#### Quality Criteria (Pass/Fail)

A. ASD sample recruited from same or similar populations (e.g. geographical area or clinical setting).
B. Established method of confirming ASD status (e.g. Autism Diagnostic Observation Schedule, Autism Diagnostic Interview, any DSM criteria).
C. No psychiatric medication use at time of assessment or steps taken to limit effect of medication on findings e.g. medication controlled for in analyses, or symptoms prior to medication use reported.
D. Established intelligence test for measuring IQ.
E. Established instrument used to asses internalising symptoms or professional diagnosis of internalising disorder.

‘Established’ refers to accepted psychometric measures in published literature, which have demonstrated reliability. Studies scoring 4 or 5 were deemed of sufficient quality and therefore appropriate for inclusion in the meta-analysis. Scores of 4 were mainly due to studies not meeting criterion C. Consequently, psychiatric medication was included as a moderator and a sub-group analysis was performed to investigate whether medication status made a difference to the overall effect size. Due to a lack of information, two studies did not meet criteria C and D (Gadow et al. 2016; Ghaziuddin and Greden 1998). They were deemed to be of a lower quality for use in this study, which was taken into account when interpreting the depression analysis results. Table 2 specifies the study characteristics and quality scores for the meta-analysis studies.

### Assessment of Heterogeneity, Publication Bias and Statistical Analyses

Statistical heterogeneity is a consequence of variability between studies, resulting from methodological differences and clinical diversity, namely differences in participants (Deeks et al. 2008). Both types of variability between the studies included in the meta-analysis were evident from the data extraction and quality assessment processes.

Firstly, IQ tests varied between studies. For nine of the 15 studies, versions of the Wechsler Scales were used. Other tests included the Stanford-Binet Intelligence Scale and Kaufman Brief Intelligence Test. Nevertheless, effect sizes obtained from these studies were considered comparable, as evidence supports high correlation between these IQ tests in people with ASD and general populations (Baum et al. 2015; Newton et al. 2008; Slate et al. 1996). All studies reported IQ or full-scale IQ scores; therefore scores were also deemed comparable.

Secondly, all studies used established methods of confirming ASD status. These methods were considered comparable, as congruence between them has been shown in an adolescent sample (de Bildt et al. 2004). Additionally, since the current study aimed to investigate how IQ relates to two internalising symptoms in a broad ASD population, varying measures for confirming ASD status were accepted. Finally, other evident sources of clinical and methodological heterogeneity were addressed using sub-group analyses.

Quantification of heterogeneity and all subsequent analyses were carried out in Comprehensive Meta Analysis (CMA) software (Version 2.0). Assessment of heterogeneity was completed for the anxiety and depression outcomes separately. Higgins’ levels of heterogeneity informed the assessment, with an I^2^ of 25%, 50% and 75% classified as low, moderate and high, respectively (Higgins et al. 2003). Results determined which type of effects model would be used in the analyses: fixed or random.

For the 13 studies with an anxiety outcome, heterogeneity was moderately high (I^2^ = 62.486%, Q(12) = 31.988, *p* < 0.01) To account for this, random effects models were used throughout all anxiety analyses. Moreover, heterogeneity results validated the decision to use sub-group analyses to test whether each of seven moderators significantly explained the variance between IQ and anxiety levels. For each moderator, I^2^ and corresponding Q measures of heterogeneity indicated the level of variance within each group under the moderator. Total Q between values (Q_between_) specified the level of heterogeneity between groups, and consequently whether the moderator had a significant effect. Since there were seven moderators for the sub-group analyses, a more stringent alpha value of 0.01 was used to indicate a significant effect. This decision was made to limit the family-wise error rate. Alternatively, a standard alpha value of 0.05 was used to determine statistical significance of the main anxiety and depression analysis results.

For the four studies with statistical data on depression, heterogeneity was low (I^2^ = 19.809%, Q(3) = 3.741). Resultantly, a fixed effects model was used for the depression outcome. Sub-group analyses were not run for depression, due to the low number of studies and low heterogeneity.

Publication bias was investigated for the studies on anxiety and depression separately, using two methods. The funnel plot method distributes effect sizes from each study around the pooled mean effect size. Asymmetrical distribution visually indicates presence of publication bias. The trim-and-fill method estimates what the effect size would be without the presence of publication bias (Duval and Tweedie 2000). This method was chosen over other alternatives, such as the fail-safe N, which has been advised against due to available methods producing widely varying estimations of bias (Sterne et al. 2008). Additionally, presence of bias due to lower reporting of non-significant effect sizes was investigated within the vote-counting procedure, which contained the studies excluded from the meta-analysis. It was predicted that the majority of the papers that did not provide effect sizes would report non-significant findings.

### Selection and Coding of Moderators

For the anxiety sub-group analyses, seven moderators were chosen based on the rationales below. Table 3 describes how data were grouped and coded.

**Table 3:** Coding system for the seven moderators in the anxiety sub-group analyses

Moderator	Coding system for groups
Person reporting anxiety	0 = Self-reported 1 = Caregiver-reported
Psychiatric medication	0 = No 1 = Yes
IQ range	0 = ≤ 70 1 = ≥ 70 2 = ≤ 70 ≤
Mean age	0 = < 13.0 years 1 = ≥ 13.0 years
Gender	0 = Male only 1 = Male and female
Effect size type	0 = *r* using continuous measurements of IQ and anxiety 1 = Effect size from IQ grouped participants 2 = Effect size from level of anxiety grouped participants 3 = Unadjusted odds ratio
DSM-IV-orientated anxiety assessment	0 = No/Unclear 1 = Yes

#### Person Reporting Anxiety

Caregiver and self-reports of anxiety were coded into two groups to test for the presence of discrepancies between the two methods of reporting anxiety, as found previously (Oswald et al. 2016), and whether this significantly affected the correlation with IQ. By incorporating this moderator, the current meta-analysis fulfilled the request of the previous meta-analysis, which asked for future studies to examine how anxiety frequency varies across informants (van Steensel et al. 2011).

#### Psychiatric Medication

Psychiatric medication use could affect reports of anxiety (Mazefsky et al. 2011). The group labelled ‘Yes’ included studies with participants on medication, as well as the study by de Bildt et al. (2005). They reported that there was no exclusion of participants based on use of medication. The group labelled ‘No’ included samples not on any medication and studies that used analysis to prove no effect of medication on any reported findings (May et al. 2015; Strang et al. 2012). Additionally, Mazefsky et al. (2011) was coded ‘No’, as participants were asked to report symptoms prior to receiving medication.

#### IQ Range

Studies were coded into three groups, depending on whether they included participants with: IQs of 70 or lower (≤ 70), IQs of 70 or higher (≥ 70), or IQs across these ranges (≤ 70 ≤). This was done to test whether the correlation found between IQ and anxiety may vary depending on the range of IQs investigated. An IQ of 70 was used as a cut off based on the ranges specified by the studies. Note that one study stated no IQ range (Rodgers et al. 2012). However, they reported that all participants had IQs within the average range. Consequently, the study was included in the ‘IQs of 70 or higher’ group. Also, in Vasa et al. (2013), despite no specified IQ range, the mean IQ (76.8) and standard deviation (23.7) were provided. Hence, it was inferred that participants with IQs above and below 70 were included and the study was coded accordingly.

#### Mean Age

By focusing on adolescence, this study anticipated limiting any significant effects of younger versus older age on the relationship between IQ and internalising symptoms. However, there is some evidence that anxiety may increase through adolescence, perhaps due to the progressive development of social awareness and self-perception (Mayes et al. 2011a, b). Resultantly, eleven of the 13 study samples in the current study had a mean age of below 13.0 years and were grouped together. The two samples with a mean age above 13.0 were categorised in a second group to test whether earlier versus later adolescence significantly affected the association between IQ and anxiety.

#### Gender

Samples that included males and females were coded separately from the male only sample. This was done to assess whether the male only sample was a significant source of heterogeneity that might impact findings on the association between IQ and anxiety.

#### Effect Size Type

The type of effect size reported in studies varied due to methodological differences in the way studies tested the correlation between IQ and anxiety. Four categories were used to test whether effect size type affected results of this meta-analysis. Studies coded ‘0′ reported correlation coefficients (*r*) calculated using continuous measurements of IQ and anxiety. Code ‘1′ studies grouped participants based on IQ scores; hence the effect sizes reflected the difference in anxiety levels in youth with higher versus lower IQs. Studies coded ‘2′ separated participants based on anxiety levels, thus effect sizes reflected the difference in IQ for those with higher versus lower anxiety. Finally, Simonoff et al. (2008) used an unadjusted odds ratio, coded for separately as ‘3’.

#### DSM-IV-Orientated Anxiety Assessment

Studies were divided into two groups according to whether the instrument used to assess anxiety was based on DSM-IV criteria. This was done to address the heterogeneity between anxiety measures and test whether it had any significant effect on results. Nine of the 13 studies used DSM-IV-orientated instruments, determining this method of grouping. The remaining four studies were coded into a second group for the following reasons. White and Roberson-Nay (2009) used the Multidimensional Anxiety Scale for Children, which is not completely congruent with DSM-IV criteria for anxiety (van Gastel and Ferdinand 2008). May et al. (2015) used the Children’s Sleep Habits Questionnaire, whilst de Bildt et al. (2005) used the Children’s Social Behaviour Questionnaire. For these instruments, it was uncertain whether the anxiety aspect (sleep anxiety and fear of changes, respectively) is assessed based on DSM criteria. Finally, Mazefsky et al. (2011) used the Revised Children’s Manifest Anxiety Scale, which does not clearly correspond with DSM-IV criteria (Lam et al. 2005).

## Results

### Anxiety: Main Analysis and Sub-Group Analyses

**Fig. 2:**
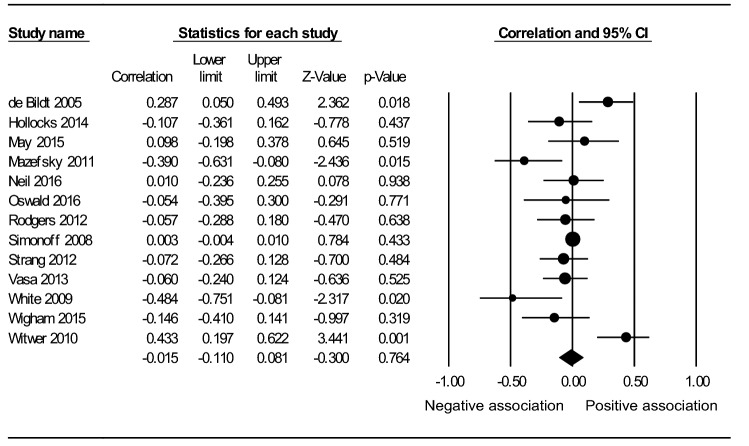
Forest plot showing a non-significant association between IQ and anxiety in adolescents with ASD, under a random effects model. The black circle for each study is proportional to the study’s weight in the anxiety analysis. The black diamond indicates the overall effect (r = − 015, p = .764)

The sample size across the 13 anxiety studies was 812. As per the forest plot (Fig. 2), the main analysis found no significant association between IQ and anxiety, under a random effects model (*r* = -0.015, *p* = 0.764). Person reporting anxiety was the only moderator significantly impacting the association between IQ and anxiety (Q_between_(1) = 9.529, *p* < 0.01). Cohen’s (1988) guidelines were used to interpret the magnitude of significant correlations: *r* > 0.50 = large, 0.50-0.30 = moderate, and 0.29-0.10 = small. Self-reported anxiety moderately negatively correlated with IQ (*r* = -0.424, *p* < 0.01), while there was no significant correlation between parent-reported anxiety and IQ. Table 4 shows the anxiety analyses results.

**Table 4:** Results of the meta-analysis examining the association between IQ and a) anxiety b) depression, in adolescents with ASD

Outcome (type of effects model)	Moderator			95% CI		Heterogeneity		
	Groups	k(N)	*r*	LL	UL	Q	I^2^ (%)	Q_between_
Anxiety (random)	Without moderators (main analysis)	13(812)	-.015	-.110	.081	31.988**	62.486	
	Person reporting anxiety:							9.529**
	Self-reported	2(55)	-.424**	-.616	-.185	0.168	0.000	
	Caregiver-reported	11(757)	.027	-.060	.113	20.546*	51.328	
	Psychiatric medication:							4.723*
	No	5(248)	-.162	-.341	.027	8.418	52.481	
	Yes	3(240)	.219	-.098	.495	11.675**	82.870	
	IQ range:							5.761
	≤ 70	1(67)	.287*	.050	.493	0.000	0.000	
	≥ 70	8(443)	-.080	-.173	.015	6.021	0.000	
	≤ 70 ≤	3(285)	.102	-.117	.313	12.138**	83.523	
	Mean age:							0.114
	< 13.0 years	11(665)	-.010	-.122	.103	31.454***	68.207	
	≥ 13.0 years	2(147)	-.059	-.220	.106	0.001	0.000	
	Gender:							0.258
	Male only	1(52)	-.107	-.361	.162	0.000	0.000	
	Male and female	12(760)	-.008	-.110	.093	31.353**	64.915	
	Effect size type:							3.509
	*r* using continuous measurements of IQ and anxiety	6(344)	-.078	-.197	.043	5.979	16.373	
	Effect size from IQ grouped participants	3(142)	.114	-.342	.527	14.306**	86.020	
	Effect size from level of anxiety grouped participants	3(214)	-.076	-.207	.058	0.077	0.000	
	Unadjusted odds ratio	1(112)	.003	-.004	.010	0.000	0.000	
	DSM-IV-orientated anxiety assessment:							0.211
	No/ unclear	4(168)	-.111	-.459	.266	17.288**	82.647	
	Yes	9(644)	-.004	-.090	.083	14.678	45.497	
Depression (fixed)	Without moderators	4(364)	.119*	.017	.219	3.741	19.809	

*CI* Confidence Interval, *k* Number of studies, *N* Sample size across studies, *r* Correlation coefficient, *LL* Lower Limit, *UL* Upper Limit, *Q & I2* Heterogeneity within groups, *Qbetween* Heterogeneity between groups

p-values * <.05 ** <.01 *** <.001

Superscript dot (.) indicates a statistically non-significant trend in the data (p<.1)

### Depression Analysis

**Fig. 3:**
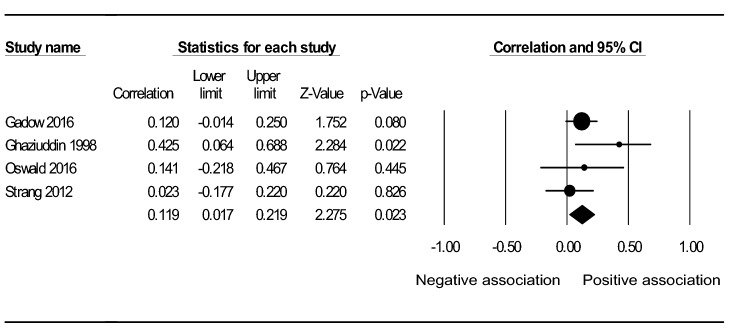
Forest plot showing a positive association between IQ and depression in adolescents with ASD, under a fixed effects model. The black circle for each study is proportional to the study’s weight in the depression analysis. The black diamond indicates the overall effect (r = .119, p < .05)

The sample size across the 4 depression studies was 364. Under a fixed effects model, a small positive correlation was found between IQ and depression (*r* = 0.119, *p* < 0.05) (Table 4). Figure 3 shows the forest plot for this analysis.

### Publication Bias

**Fig. 4:**
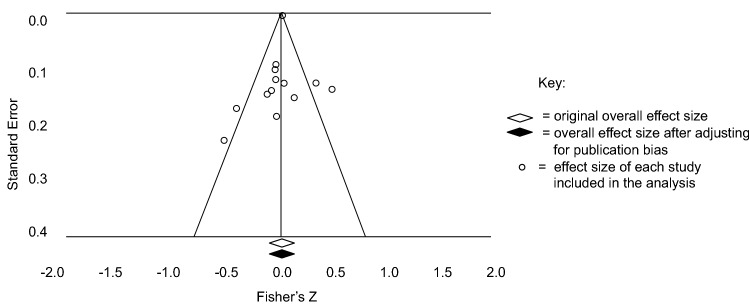
Funnel plot for the anxiety publication bias analysis under a random effects model

**Fig. 5:**
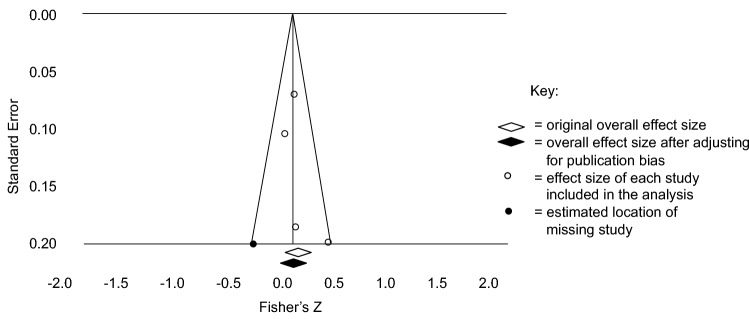
Funnel plot for the depression publication bias analysis under a fixed effects model

Asymmetrical distribution of effect sizes in the funnel plots for both anxiety and depression indicated presence of publication bias. The trim-and-fill method provided estimations of overall effects after adjusting for publication bias. Anxiety remained non-significantly correlated with IQ (*r* = 0.044, 95% CI [-0.056, 0.143]). For depression, the correlation with IQ became non-significant after adjustment (*r* = 0.094, 95% CI [-0.052, 0.192]). Figures 4 and 5 display these results. Finally, 8 of the 11 findings without effect sizes were reported as non-significant.

### Vote-counting Results

Table 5 shows the vote-counting results of the studies excluded from the meta-analysis. Eight of the 11 findings without effect sizes were reported as non-significant.

**Table 5:** Vote-counting results of the studies excluded from the meta-analysis

Internalising symptom	Effect size reported	Correlation with IQ	Number of studies	Studies
Anxiety	Yes	Positive	1	Hallett et al. 2013^a^
Anxiety	Yes	Negative	1	Hallett et al. 2013^a^
Anxiety	No	Positive	1	Gotham et al. 2013
Anxiety	No	Negative	0	n/a
Anxiety	No	Non-significant	3	Gjevik et al. 2011; Kerns et al. 2014; Kim et al. 2000
Depression	No	Positive	1	Vickerstaff et al. 2007
Depression	No	Negative	0	n/a
Depression	No	Non-significant	2	Gjevik et al. 2011; Mazzone et al. 2013
Grouped	Yes	Positive	2	Taylor and Henninger 2015; van Steensel et al. 2013
Grouped	Yes	Negative	0	n/a
Grouped	No	Positive	0	n/a
Grouped	No	Negative	1	Rosa et al. 2016
Grouped	No	Non-significant	3	Bitsika et al. 2016; Meyer et al. 2006; Schroeder et al. 2011

^a^Included twice due to positive and negative correlations found between IQ and anxiety, depending on the anxiety subtype analysed and whether the anxiety was parent or self-rated

## Discussion

This study aimed to synthesise the current evidence to distinguish the overall direction of the association between IQ and two prevalent internalising symptoms, depression and anxiety, in adolescents with ASD. It also sought to highlight methodological factors contributing to inconsistent findings in the existing literature, in order to assist in achieving the first aim, as well as guide future research. A meta-analysis was used to evaluate the relationship between IQ and (a) anxiety (b) depression, using effect sizes provided by 13 and four studies, respectively. Before taking into account moderating factors, no significant relationship was found between IQ and anxiety (*r* = -0.015, *p* = 0.764). However, when only self-reported anxiety was considered, anxiety was moderately higher in less intelligent youth (*r* = -0.424, *p* < 0.01). This sub-group analysis result reflects the negative association found by a previous meta-analysis (van Steensel et al. 2011). Furthermore, this study found a significant effect showing higher levels of depression in more intelligent youth (*r* = 0.119, *p* < 0.05), consistent with a recent study of 1272 children and adolescents with ASD (Greenlee et al. 2016).

### Publication Bias

The trim-and-fill method estimated that the associations between IQ and the internalising symptoms would be non-significant without the presence of publication bias (Figs. 4, 5). These results are unsurprising, as non-significant findings are less likely to be published and therefore included in this meta-analysis (Egger and Smith 1998). To put this issue into perspective, the studies excluded from the meta-analysis for not providing effect sizes were investigated within the vote-counting procedure. As predicted, the majority of these studies reported non-significant associations. Vote-counting results are discussed alongside the meta-analysis findings.

### Anxiety Results

As expected, vote-counting showed that most of the studies excluded for lacking effect size data reported a non-significant association between anxiety and IQ (Table 5), consistent with the non-significant meta-analysis result. However, studies with lower sample sizes may have found effects that were too weak to detect and therefore deemed non-significant. Had all of the studies reported effect sizes, they might have significantly affected the anxiety analysis result by contributing towards a positive or negative correlation with IQ.

Combining the findings of the anxiety analysis and the vote-counting procedure, our results indicate that, before taking into account any moderating factors, IQ is unrelated to anxiety levels. However, the sub-group analyses identified a significant negative relationship between self-reported anxiety and IQ, providing a potential methodological reason for the non-significant result of the main anxiety analysis, as discussed below.

### Sub-Group Analyses

Seven moderators were tested to determine whether they significantly affected the association between IQ and anxiety (Table 4).

Person reporting anxiety: Self versus caregiver-reported anxiety was the only significant moderator in the sub-group analyses [Q_between_(1) = 9.529, *p* < 0.01]. This finding supports the existing concern of discrepancies between parent and self-reported internalising symptoms (Sterling et al. 2015).

Self-reported anxiety was moderately greater in adolescents with lower IQs (*r* = -0.424, *p* < 0.01), challenging the notion that those with lower intelligence are less able to acknowledge and communicate symptoms than more intelligent youth (Caamaño et al. 2013). It could also be that more intelligent individuals experience less anxiety, accurately reflected in their reporting. The result highlights the importance of acknowledging variations in individual abilities within ASD, rather than making assumptions based on IQ levels. While some adolescents with reduced intellectual abilities may be unable to communicate their emotions, this study shows others are capable. Consequently, caregivers and clinicians should establish the ability of an individual to self-identify symptoms, irrespective of their IQ, when assessing their mental health.

Conversely, caregiver-reported anxiety and IQ were not associated. It is possible that caregivers both underestimate and overestimate anxiety levels in youth with ASD, obscuring the correlation with IQ. It is well established in literature that parents often underreport the anxiety experienced by their children (Cosi et al. 2010; Muris et al. 1999). By contrast, anxious parents have been shown to over-report anxiety in children (Bernstein et al. 2005). Furthermore, caregivers may overestimate or under-report anxiety by confusing symptoms with ASD traits, such as communication deficits and social avoidance (Davis et al. 2011; Kuusikko et al. 2008). Also, parents of individuals with poorer abilities to communicate feelings may struggle to accurately report anxiety symptoms, particularly if external signs do not accompany them. Caregiver under-reporting in less intelligent youth may explain why higher self-reported anxiety, but not caregiver-reported anxiety, was found to relate to a lower IQ in the current meta-analysis. In line with this suggestion, in the study by Hallett et al. (2013), greater anxiety was associated with higher IQs when parent-rated, but when self-rated, it was associated with lower IQs. Thus, while measuring different anxiety subtypes might explain the divergence in findings, using different perspectives to report symptoms provides another explanation.

Ultimately, clinical heterogeneity between caregivers and amongst youth with ASD is inevitable and will affect the level of accuracy to which any particular caregiver reports anxiety. The current results suggest that caregiver and self-reports should be used in combination to construct a more representative symptom profile. Additionally, parents should be cautious of underestimating anxiety levels in adolescents with lower IQs.

#### Psychiatric Medication

Psychiatric medication use did not significantly influence the relationship between IQ and anxiety. Mazefsky et al. (2011) suggested that psychiatric medication use limits studies investigating psychiatric problems. However, this finding implies that including individuals on psychiatric medication is not a major concern when assessing how IQ relates to anxiety in individuals with ASD. A large proportion of the ASD population take psychiatric medication (Mazefsky et al. 2011). Hence, the result is favourable to this meta-analysis and future studies, as excluding medicated individuals would restrict the range of youth eligible for investigation and reduce the generalisation of results to the broader ASD population.

#### IQ Range

The association between anxiety and IQ did not significantly differ depending on whether studies included participants with: IQs of 70 or lower, IQs of 70 or higher, or IQs across these ranges [Q_between_(2) = 5.761, *p* < 0.1]. This contradicts the suggestion that studies analysing a wider IQ range might be more likely to find a significant relationship with emotional problems (Fung et al. 2015).

Nevertheless, the results indicate a potential non-linear trend in the data, also found by the previous meta-analysis (van Steensel et al. 2011). For adolescents with IQs of 70 or lower, a small effect was found such that anxiety increased with IQ (*r* = 0.287, *p* < 0.05). Conversely, for those with IQs of 70 or above, there was a negative trend: anxiety increased as IQ decreased (*r* = -0.080, *p* < 0.1). In other words, within these two groups, anxiety was higher in participants with IQs closer to 70. No significant correlation was found for the group containing IQs above and below 70. Collectively, these findings tentatively indicate a quadratic relationship between IQ and anxiety.

One explanation is that it is possible for individuals with IQs nearing 70 or ‘borderline’ intellectual disability to be susceptible to risk factors for anxiety related to a lower IQ, as well as those associated with a higher IQ. For instance, they may have inadequate adaptive coping strategies and communication skills, which have been suggested to relate to higher anxiety in those with lower intelligence (Crabbe 2001; Pickersgill et al. 1994). Yet, a lack of severe cognitive impairment increases their likelihood of integrating with typically developing youth and facing the challenge of keeping up academically and socially (Anderson et al. 2011). They may also receive less social and emotional support than youth with more explicit intellectual disabilities, who may be prioritised by local services (Taylor and Seltzer 2011) or more closely monitored by caregivers. Moreover, self-awareness of deficits has been suggested to cause anxiety in people with ASD (Caamaño et al. 2013). Youth with borderline intelligence may have greater deficits than more intelligent individuals, coupled with more self-awareness than those with lower IQs, causing higher anxiety levels overall.

It should be noted that an IQ of 70 was used as a cut off based on the ranges used by studies; it is not necessarily the score at which the association changes if a non-linear correlation exists. The previous meta-analysis suggested an IQ between 70–87 was associated with higher anxiety, but only under a fixed effects model (van Steensel et al. 2011). Overall, the tentative findings of the current and previous meta-analyses provide reason for further investigation into the possibility of a middle ground IQ range at which individuals may be more susceptible to anxiety.

#### Mean Age

A sample mean age above versus below 13.0 years did not significantly affect the relationship between IQ and anxiety. Focusing on an adolescent population could have minimised any effect of younger versus older age on this investigation. Furthermore, during adolescence, the timings of factors that could induce anxiety vary from one individual to the next, for instance, the age at which youth reach puberty or develop self-consciousness. Age may, therefore, have no overall distinct association with anxiety in adolescents with ASD, as found by Strang et al. (2012). However, the small sample sizes limit the strength of this finding.

#### Gender

The association between IQ and anxiety did not significantly differ for the male only sample compared to the mixed gender samples. This is unsurprising, as every sample included in the meta-analysis was over 50% male. This gender imbalance is representative of the higher rate of identification of ASD in males compared to females (Fombonne 2009). Nevertheless, more research on how IQ relates to anxiety in females with ASD would allow clarification of whether current findings are relevant across genders.

#### Effect Size Type

Type of effect size, related to the way in which studies grouped participants, did not significantly affect the association between IQ and anxiety. This finding validates the decision of this meta-analysis to include studies with varying methodologies for measuring the association between IQ and anxiety.

#### DSM-IV-Orientated Anxiety Assessment

The relationship between IQ and anxiety did not significantly differ depending on whether or not a DSM-IV-orientated instrument for anxiety assessment was used. This finding suggests that measurements of anxiety using DSM-IV-orientated instruments are comparable to those that were potentially based on other criteria. It validates the decision of this meta-analysis to include studies using either type of instrument. Nonetheless, this was only one method of categorising heterogeneity between measures. Instruments could still vary in their ability to accurately assess anxiety in individuals with ASD, particularly since the majority are designed for the general population, such as the Child Behaviour Checklist (Vasa et al. 2013). This could affect accuracy, as signs of anxiety in those with ASD may differ from those shown by typically developing youth (Stern et al., 2014). Furthermore, questions that allow detection of anxiety in typically developing youth may not be appropriate for those with ASD, as found by Witwer and Lecavalier (2010). They administered the Children’s Interview for Psychiatric Symptoms, which asked some inapplicable questions, such as those relating to school grades. Resultantly, educational setting might have affected the measurement of anxiety obtained. More research into which instruments are most reliable at measuring anxiety in individuals with ASD would benefit the assessment of youth in future studies and in clinical practice.

Finally, although the current study was unable to moderate for anxiety subtype due to a lack of studies with anxiety subtype data, it is worth noting that the term anxiety encompasses several disorders, such as generalised anxiety disorder, panic disorder, post traumatic stress disorder, social phobia and obsessive–compulsive disorder. The previous meta-analysis found that the relationship between IQ and anxiety differed, depending on the anxiety subtype analysed (van Steensel et al. 2011). It could be that certain sources of anxiety are associated more closely with particular intelligence levels. For example, Hallett et al. (2013) observed greater parent-rated social anxiety in adolescents with higher IQs and greater self-rated separation anxiety in those with lower IQs. They suggested the former might be because more intelligent youth have a better social understanding and more concern regarding fitting in among peers. Less intellectually able youth may require more support from caregivers, explaining the higher anxiety when separated from them.

Nevertheless, even when the associations between IQ and anxiety subtypes in individuals with ASD are investigated, findings vary. Contrary to the above, higher social anxiety in less intelligent youth has been reported, possibly due to a lower awareness of social cues and greater difficulty adapting to social situations (Oswald et al. 2016; van Steensel et al. 2011). Also, a higher IQ has been associated with increased separation anxiety (van Steensel et al. 2011). To further build on the understanding of how IQ relates to anxiety, further investigation into the associations between IQ and anxiety subtypes, as well as the underlying mechanisms, is required.

### Depression Results

For depression, only positive or non-significant associations with IQ were reported by studies in the vote-counting procedure (Table 5). These results are consistent with the lack of negative correlation shown by the meta-analysis, which found greater levels of depression in youth with higher IQs (*r* = 0.119, *p* < 0.05) and no association after adjustment for publication bias. Nevertheless, the effect that the findings of the excluded studies might have had on the meta-analysis, had they reported effect sizes, is uncertain.

Overall, results suggest that adolescents with lower IQs may be less susceptible to depression. Potential explanations include these individuals having more explicit disabilities, therefore receiving more robust social and emotional support than those with higher IQs, who are left to be more independent. Also, youth with lower IQs with a reduced self-awareness of deficits may be protected from low self-esteem and associated depression (Mayes et al. 2011a, b). Conversely, adolescents with autism who are more able to perceive themselves as different from their peers have been shown to experience greater depression (Hedley and Young 2006). The current results reiterate the importance of adequate emotional support for youth with autism, including those with higher IQs.

However, the results also support the argument that depression is more accurately reported in the presence of a higher IQ. This could result from greater observance of symptoms by clinicians and caregivers, or a better ability to self-report feelings (Caamaño et al. 2013; Ghaziuddin and Greden 1998). Additionally, all but one study in this analysis used parent-reported depression. Thus, the inference drawn from the anxiety analysis that parents may underestimate symptoms in less intelligent youth might also be relevant to depression, although the current study cannot conclude this. A better insight into how depression can be detected in less intelligent youth is required to determine whether they may be at a similar risk to those with higher IQs.

### Grouped Internalising Symptoms Results

Vote-counting results showed that, across the six studies, there was no overall agreement in the direction of the association between IQ and grouped internalising symptoms (Table 5). These results, alongside the meta-analysis results, validate the decision to analyse anxiety and depression separately, by supporting the theory that relationships between different internalising symptoms and IQ in individuals with ASD may vary. The answer to why these relationships can differ is yet to be determined. One suggestion is that anxiety might be somewhat alleviated through communication, particularly if the source of anxiety is known. Discussing worries and fears might be easier for those with a higher IQ (Pickersgill et al. 1994), causing them to report lower levels of anxiety, as found in this meta-analysis. Conversely, depressive symptoms may not be lessened by communication in the same way, especially if there is no external trigger to talk through. This, amongst other factors, may cause more intelligent youth to be at a greater risk of depression than anxiety. This is in line with the current findings of high levels of depression in the presence of a higher IQ. With the directions of the associations between IQ and the two prevalent internalising symptoms in adolescents with ASD now further established, a clearer understanding of the mechanisms underlying the relationships is needed to shed light on why they may differ.

## Limitations

There are several limitations in this study. Firstly, anxiety subtypes (such as generalised anxiety disorder, panic disorder, post traumatic stress disorder, social phobia and obsessive–compulsive disorder) could not be assessed and some of the moderator groups were small. Secondly, having only four depression studies meant sub-group analyses were not possible. If the non-significant effect of psychiatric medication on anxiety analysis results was extrapolated to depression, the two papers that did not meet the current criteria for inclusion would have otherwise been included. As a result, the anxiety sub-group analyses findings may not be the same for depression.

Thirdly, the existing literature lacks studies focusing on groups with a low IQ; only four out of the 13 anxiety studies included in this meta-analysis stated that they incorporated youth with below-average IQ. A further 3 studies did not report IQ ranges (Table 2); one such study reported that all participants had IQs within the average range (Rodgers et al. 2012), while White and Roberson-Nay (2009) used a mean IQ of 92.24. However, Vasa et al. (2013), reported mean IQ of 76.8 suggesting youth with lower IQ were included in the study. Individuals with autism and intellectual disability is an understudied population within the autism spectrum (Jack and Pelphrey 2017). Similarly, when studying ASD one of the limitations of many studies, including those incorporated in the current meta-analysis, is the inherent sex bias where more males are diagnosed with this condition compared to females (Lai et al. 2013).

Finally, due to a lack of previous studies using solely adolescent-aged samples, this meta-analysis had to implement an inclusion criterion whereby studies with mean age 10–19 were accepted. However, this study provides an important start in addressing this research gap and contributing to the understanding of mental health problems commonly faced within the adolescent autism population specifically, which future research must continue to do.

### Strengths and Key Findings, Including Implications for Future Research and Clinical Practice

Fulfilling its primary aim, this study synthesises the current evidence to distinguish the overall direction of the association between IQ and two prevalent internalising symptoms, anxiety and depression, in adolescents with ASD. The results, summarised below, provide a clearer understanding of the relationship between these symptoms and IQ. It is hoped that the findings from this study will contribute towards allowing youth to be more readily identified and supported in clinical practice, educational settings and at home.

In agreement with a study of 1,272 youth with ASD, this meta-analysis has found that a higher IQ relates to greater levels of depression (Greenlee et al. 2016). Thus, in clinical practice, due to an increased risk of depression, care should be taken to ensure that youth with ASD without intellectual disabilities are still provided with adequate social and emotional support.

The anxiety meta-analysis indicates that youth with a lower IQ experience higher anxiety when self-reported. The previous meta-analysis also found a negative correlation between IQ and anxiety in ASD (van Steensel et al. 2011). However, a limitation of that study was the insufficient data to test whether informant (parent versus child) moderated the relationship between anxiety and ASD. The sub-group analyses in the current study fulfils the request by Van Steensel et al (2011) asking future research to examine how anxiety frequency varies across informants. Furthermore, as per the second aim of the study, it highlights a key factor contributing to discrepancies in existing evidence on this topic: use of self or caregiver-reported symptoms. In line with tentative findings from Van Steensel et al (2011), the current sub-group analyses also showed that individuals with ASD and ‘borderline’ intellectual disability may be particularly susceptible to anxiety Within clinical practice, it is important that the ability of adolescents with ASD to self-identify and report internalising symptoms is established, irrespective of their IQ, when their mental health is being assessed. Parents and clinicians should be cautious of underestimating anxiety in individuals with a lower IQ, who may themselves report significant symptoms. Overall, the congruity of the results from the current anxiety and depression meta-analyses with large previous studies aid in establishing the previously unclear directionality of the relationships between internalising symptoms and IQ in ASD.

As discussed above, the study has shown a significant positive relationship between depression and IQ, and a significant negative relationship between self-reported-anxiety and IQ, proving a need to assess different internalising symptoms separately, a strength of the current meta-analysis. To add further clarity, future studies should separate anxiety into subtypes.

Finally, this meta-analysis has highlighted understudied groups: future research should focus specifically on females, the adolescent age group and youth with a low IQ to ensure they are not neglected, as we continue to improve our understanding and treatment of the mental health concerns prevalent within the adolescent autism population.

## Appendix A

### Complete List of the Search Terms Used Across the Databases: MEDLINE, PsycINFO and Scopus

#### Medical Subject Headings

Asperger syndrome OR autism spectrum disorder OR autistic disorder OR autistic thinking OR exp autism spectrum disorders AND anxiety disorders OR exp anxiety disorders OR agoraphobia OR anxiety, separation OR obsessive–compulsive disorder OR panic disorder OR phobic disorders OR depressive disorder OR depressive disorder, major OR depression (emotion) OR major depression OR sadness AND adolescent development OR child development OR childhood development OR adolescent OR child AND intelligence tests OR stanford-binet test OR wechsler scales OR intelligence OR intelligence quotient OR exp intelligence measures OR intellectual development disorder OR intellectual disability.

Note: ‘exp’ refers to exploded Medical Subject Headings, which include all the narrower search terms in the hierarchical list.

#### Keywords (in title or abstract)

Autis* OR asperger AND anxiety OR anxious OR fear OR depress* OR sad OR withdraw* OR internalising OR internalizing AND adolescen* OR child* OR youth OR teenage* AND IQ OR “intellectual ability” OR ID OR “intellectual disability” OR “cognitive ability” OR “learning disability” OR “mental retardation”.

Note: the asterisk (*) indicates that the search term includes the stem specified, but allows for different endings.

## Appendix B

### Rationales for Choice of Effect Size Extracted From Studies Reporting more than one Effect size

Witwer and Lecavalier (2010): Four chi-squared statistics for the association between IQ and anxiety were reported. Three statistics related to generalised anxiety disorder sub-scores for: ‘worry more’, ‘hard to relax when worried’ and ‘trouble letting go of worries’. One statistic related to a ‘fear keeps from school’ phobia score. The effect size for ‘worry more’ was chosen, as it was deemed to best reflect the definition of anxiety.

Oswald et al. (2016): For the anxiety analysis, the ‘total anxiety’ score was chosen as the outcome over anxiety subtype scores. Also, the parent-reported total anxiety score was used instead of the self-reported score. These decisions were both made to increase the homogeneity of studies in the meta-analysis, as most studies used parent-reports of any anxiety. Similarly, for the depression analysis, parent-reported depression was used rather than self-reported depression to increase homogeneity of studies in this analysis.

Simonoff et al. (2008): The male and female statistic was chosen instead of the male only statistic, due to the larger sample size and for consistency with most studies included in the anxiety analysis.
